# Clinical Manifestations Associated with Overweight/Obesity in Puerto Ricans with Fibromyalgia Syndrome

**DOI:** 10.1155/2016/1379289

**Published:** 2016-01-18

**Authors:** Ruth M. Fred-Jiménez, Mariangelí Arroyo-Ávila, Ángel M. Mayor, Grissel Ríos, Luis M. Vilá

**Affiliations:** ^1^Division of Rheumatology, Department of Medicine, University of Puerto Rico, Medical Sciences Campus, San Juan, PR 00936, USA; ^2^Retrovirus Research Center, Department of Internal Medicine, Universidad Central del Caribe School of Medicine, Bayamón, PR 00960, USA

## Abstract

*Objective*. To determine the clinical manifestations associated with overweight/obesity in Hispanics from Puerto Rico with fibromyalgia syndrome (FMS).* Methods*. A cross-sectional study was performed in 144 patients with FMS (per American College of Rheumatology (ACR) classification criteria). Sociodemographic features, FMS-related symptoms, tender points (per ACR criteria), comorbidities, and FMS treatment were examined. BMI was calculated and patients were grouped into two categories: BMI ≤ 24.9 kg/m^2^ (nonoverweight/obese) and BMI ≥ 25 kg/m^2^ (overweight/obese). Bivariate and multivariate analyses were used to evaluate differences between the study groups.* Results*. The mean (standard deviation (SD)) age of patients was 50.2 (9.9) years; 95.1% were females and 75.7% were overweight/obese. In the bivariate analysis, overweight/obese patients were more likely to have self-reported memory impairment, anxiety, shortness of breath, and urinary frequency than nonoverweight/obese patients. In addition, the tender point count was higher in the overweight/obese group. In the logistic regression analyses, self-reported memory impairment and urinary frequency differences remained significant after adjusting for confounding variables.* Conclusion*. In this population of Puerto Ricans with FMS, overweight/obese patients experienced more FMS-related manifestations than nonoverweight/obese individuals. However, prospective studies are needed to confirm these associations and to elucidate if weight reduction interventions could favorably impact the severity of FMS.

## 1. Introduction

Fibromyalgia syndrome (FMS) occurs predominantly in women and is characterized by diffuse musculoskeletal pain, tiredness, sleep disorder, headaches, and cognitive impairment, among several other manifestations [1, 2]. The exact pathogenesis of FMS remains to be elucidated as well as the contributing factors associated with the severity of symptoms [3–6]. Obesity and overweight status are common comorbidities in FMS patients [3, 4, 7]. Obesity is associated with increased sensitivity to pain, poor health status, and a low health-related quality of life [8–10]. Because obesity and FMS share some clinical features it is not surprising that some studies have found that FMS patients with obesity have worse symptoms including higher pain perception, lower physical strength, poorer sleep quality, and lower self-reported quality of life [3, 4, 6]. In addition, obesity was found to be an independent risk factor for FMS in a longitudinal cohort of Norwegian women [11]. The full spectrum of FMS manifestations has not been fully studied in overweight/obese FMS patients. Thus, we sought to examine the clinical manifestations associated with overweight/obesity in a group of Puerto Ricans with FMS.

## 2. Methods

### 2.1. Patient Population

A cross-sectional study was performed in adult (≥21 years) Puerto Rican patients with FMS, as defined by the 1990 American College of Rheumatology (ACR) criteria [12]. Patients younger than 21 years, with a history of drug or alcohol abuse, and those with secondary FMS due to autoimmune rheumatic conditions such as systemic lupus erythematosus, rheumatoid arthritis, Sjögren's syndrome, inflammatory muscle disease, mixed connective tissue disease, and seronegative inflammatory arthritis were excluded. Consecutive patients were enrolled between December 2008 and December 2009 at the Rheumatology Clinics of the University of Puerto Rico Medical Sciences Campus (UPR-MSC) in San Juan, Puerto Rico, and at 2 private rheumatology practices located in San Juan, Puerto Rico. All were of Puerto Rican ethnicity (self and 4 grandparents). This study was approved by the University of Puerto Rico Medical Sciences Campus Institutional Review Board.

At study visit, clinical investigators performed a complete history and physical examination. All clinical investigators were rheumatologists certified in internal medicine and rheumatology by the American Board of Internal Medicine. They completed a structured clinical form for each patient to gather data about demographic parameters, lifestyle behaviors, FMS clinical manifestations, comorbidities, and pharmacologic treatment. This form was developed by the UPR-MSC Rheumatology Division to evaluate clinical information uniformly and to allow assessing FMS outcomes at each patient's visit. If necessary, medical records of FMS patients were reviewed to gather further information about previous clinical manifestations, comorbidities, and pharmacologic therapy.

### 2.2. Variables

Sociodemographic features, health-related behaviors, FMS-related symptoms, tender points (per ACR criteria), comorbidities, and FMS treatment were examined. Variables from the demographic domain included age, gender, and years of education. Disease duration was defined as the time interval between FMS diagnosis and study visit. Health-related behaviors including cigarette smoking, alcohol consumption, and exercise were also evaluated. The latter was defined as regular participation in physical activity as part of a personal fitness plan.

FMS manifestations included current (within the last month) FMS-related symptoms and tender points. The following symptoms were determined: tiredness, anorexia, weight loss, insomnia, headaches, anxiety, depression, memory impairment, arthralgias, joint swelling, myalgias, morning stiffness, shortness of breath, constipation, diarrhea, urinary frequency, paresthesias, Raynaud's phenomenon, sicca symptoms, and dysmenorrhea. Tender points were assessed as described in the 1990 ACR classification for FMS. The examined sites (9 pairs) were the occiput at the suboccipital muscle insertions, low cervical area at the anterior aspects of the intertransverse spaces at C5–C7, trapezius muscle at the midpoint of the upper border, supraspinatus muscle at origins, second rib at costochondral junctions, 2 cm distal to the lateral epicondyle, upper outer quadrant of the buttocks, posterior to the greater trochanteric prominence, and knees at the medial fat pad proximal to the joint line. Tender point data was reported as a count of positive tender point sites. The maximum score for this assessment is 18.

Body mass index (BMI) was calculated and patients were grouped into two categories: BMI ≤ 24.9 kg/m^2^ (nonoverweight/obese) and BMI ≥ 25 kg/m^2^ (overweight/obese). Cumulative comorbidities assessed included osteoarthritis, cervical spine disease, lumbar spine disease, osteoporosis, peripheral neuropathy, hyperthyroidism, hypothyroidism, hypertension, hyperlipidemia, type 2 diabetes mellitus, coronary artery disease, stroke, bronchial asthma, chronic obstructive pulmonary disease, and malignancy. The current (within the last month) and past use (until a month prior to the evaluation date) of the following medications for FMS were determined: tricyclic antidepressants (TCA), serotonin selective reuptake inhibitors (SSRI), serotonin-norepinephrine reuptake inhibitor (SNRI), anticonvulsants, benzodiazepines, muscle relaxants, nonsteroidal anti-inflammatory drugs (NSAIDs), cyclooxygenase-2 (COX-2) inhibitors and opioids. Psychotherapy, physical therapy, and acupuncture were also ascertained.

### 2.3. Statistical Analysis

Descriptive statistics were calculated for all variables. Contingency tables were generated to assess the relationships of demographic and clinical characteristics with BMI groups. Comparisons of categorical variables were performed using chi-square or Fisher's exact test if the expected frequency in a cell was less than 5. Continuous variables were analyzed with Student's *t*-test or the Mann-Whitney *U* test if variables were not normally distributed. Variables with a *p* value < 0.05 in the bivariate analyses plus age, gender, and disease duration were entered into the logistic regression analyses. For the interpretation of results, *p* values below 0.05 were considered statistically significant. Statistical analyses were performed with Stata 11.0 (Stata Corporation, College Station, Texas).

## 3. Results

A total of 144 FMS patients were evaluated. One hundred and thirty-seven (95.1%) were women. Thirty-five patients (24.3%) were classified as nonoverweight/obese (BMI ≤ 24.9 kg/m^2^) and 109 patients (75.7%) were classified as overweight/obese (BMI ≥ 25 kg/m^2^). As shown in Table 1 there were no significant differences between the groups in terms of sociodemographic characteristics and health-related behaviors.

Table 2 shows FMS-related symptoms and tender points among the study groups. Overweight/obese patients were more likely to have self-reported memory impairment (82.6% versus 62.9%, *p* = 0.015), anxiety (82.6% versus 65.7%, *p* = 0.035), shortness of breath (62.4% versus 42.9%, *p* = 0.042), urinary frequency (61.5% versus 31.4%, *p* = 0.002), and higher median tender point count (16 (IQR 12–18) versus 18 (IQR 14–18), *p* = 0.043) than nonoverweight/obese patients. No significant differences were seen for tiredness, anorexia, weight loss, insomnia, headache, depression, arthralgias, joint swelling, myalgias, morning stiffness, constipation, diarrhea, Raynaud's phenomenon, sicca symptoms, and dysmenorrhea.

Table 3 shows selected comorbid conditions in our FMS population. Overweight/obese patients were more likely to have type 2 diabetes mellitus (12.8% versus 0%, *p* = 0.016) than nonoverweight/obese patients. No significant difference was seen for other comorbidities. The current and past FMS therapies are shown in Table 4. Current use of anticonvulsants (gabapentin and pregabalin) (53.2% versus 31.4%, *p* = 0.025) and past exposure to benzodiazepines (42.2% versus 22.9%, *p* = 0.040) were observed more commonly in overweight/obese patients. No significant differences were seen for other pharmacologic therapies (antidepressants, muscle relaxants, NSAIDs, COX-2 inhibitors, and opioid) or nonpharmacologic therapies (psychotherapy, physical therapy, acupuncture, and chiropractic therapy).

In the logistic regression analyses, memory impairment and urinary frequency differences remained significant after adjusting for age, gender, disease duration, type 2 diabetes mellitus, current use of anticonvulsant medication, and past use of benzodiazepines (Table 5). Other manifestations associated with overweight/obesity in the bivariate analysis such as shortness of breath, anxiety, and tender point count did not reach statistical significance in the multivariate analysis.

## 4. Discussion

In this cross-sectional study of 144 Puerto Ricans with FMS, we studied the association of BMI with clinical manifestations of FMS. We found that overweight/obesity was associated with self-reported memory impairment, anxiety, shortness of breath, urinary frequency, and larger number of tender points. Self-reported memory impairment and urinary frequency retained significance in the multivariate analysis after adjusting for confounding factors.

In agreement with previous studies we found a high prevalence of overweight/obesity in our FMS population [3, 4]. Seventy-six percent of our FMS patients had a BMI ≥ 25 kg/m^2^. However, the proportion of overweight/obese patients in our study is similar to that reported for the general population of Puerto Rico [13–15]. A recent local survey of 6,025 Puerto Ricans adults found that 70% of respondents were overweight or obese [13].

Neurocognitive symptoms are commonly reported by FMS patients and these have a major impact on their quality of life [16–18]. Our study showed that overweight/obese patients reported memory impairment more frequently than nonoverweight/obese FMS patients. These results are consistent with other works. In a case-control study comparing FMS patients with and without cognitive dysfunction, Fava et al. reported associations between cognitive impairment, obesity, and insulin resistance [19]. In another case-control study, Grace et al. reported that FMS patients with perceived memory deficits frequently had more levels of anxiety and pain based on self-reported questionnaires [16]. Interestingly, in our study overweight/obese patients, in addition to reporting greater memory impairment, also had more self-reported anxiety compared to those who were nonoverweight/obese. Similarly, Aparicio et al. found that obesity was associated with anxiety and depression in a group of 175 Spanish women with FMS [7]. In our analysis, however we found no association between BMI and depression.

FMS patients commonly report urogenital disorders [20, 21]. In our study, urinary frequency was more common among overweight/obese FMS patients. This finding is not surprising as the association between high BMI and female urogenital disorders has been previously reported in the general adult population [22, 23]. In a 5-year longitudinal study, Handa et al. observed strong associations between obesity and symptoms of overactive bladder and stress incontinence among a group of parous women [22]. Similarly, McGrother et al. found that obesity was associated with new-onset overactive bladder in a group of 3,411 women in the United Kingdom [23]. Thus, it is not unexpected to find similar associations in FMS populations.

In the bivariate analysis we found that overweight/obese patients had a higher tender point count than nonoverweight/obese patients. In addition, these patients used anticonvulsants more frequently and reported a more frequent past use of benzodiazepines than FMS patients in the nonoverweight/obese group. The relationship between BMI and number of tender points has been previously observed in other studies [3, 5]. Interestingly, obesity has also been linked to increased perception of pain in the general population [9]. In a cross-sectional study of 3,637 patients residing in the Southeastern Unites States, a linear association between BMI and patient-reported pain was observed [9]. Obese patients were more likely to report pain after adjusting for age, gender, race, education level, and presence of health insurance. These findings could explain the association between BMI and pain perception in FMS. Moreover, it has been reported that weight reduction, by either conservative measures or bariatric surgery, can decrease pain scores and number of tender points in obese patients with FMS [24, 25].

Obesity has been associated with multiple comorbid conditions [10]. We studied the associations between BMI and selected comorbidities common to FMS patients. As expected, we found that patients who were overweight or obese had more frequently type 2 diabetes mellitus than nonoverweight/obese patients. Conversely, no significant differences were observed for other conditions generally associated with obesity, such as arterial hypertension, hyperlipidemia, coronary artery disease, bronchial asthma, and osteoarthritis [10, 26]. Even though overweight/obese patients with FMS had significantly more self-reported anxiety, no difference was seen between study groups in physician-diagnosed anxiety disorder.

Despite the fact that the association between obesity and FMS clinical manifestations has been consistently observed in several studies including ours [3, 4, 19, 24, 25] the exact mechanism underlying this phenomenon has not been clearly described. One possible mechanism may be related to adipocytokines, particularly proinflammatory cytokines produced by adipose tissue. These cytokines have been associated with various inflammatory conditions and malignancies [27, 28]. Proinflammatory cytokines such as interleukin-6, interleukin-8, and tumor necrosis factor-*α* have been demonstrated in both obese and FMS patients [29–31]. In addition, both conditions are associated with dysregulations of the hypothalamic-pituitary-adrenal axis and with autonomic dysfunction and sympathetic hyperactivity [32–35]. Obesity is also associated with impaired health-related quality of life, decreased physical function, and short sleep duration, which are also predominant in FMS [8, 36, 37]. Studies have shown that scheduled exercise training is beneficial in improving various FMS symptoms [38]. The fact that overweight and obese patients typically do not adhere to a scheduled exercise program might contribute to the elevated symptom perception experienced in this group compared to nonoverweight/obese patients with FMS.

We recognize that our study has some limitations. First, because of its cross-sectional nature no causal inferences can be made regarding BMI and FMS manifestations, only associations. Second, no validated questionnaires such as Fibromyalgia Impact Questionnaire were used to assess variables and most of the clinical manifestations were patient-reported. Nonetheless, patient-reported manifestations and patient-centered outcomes have an important clinical value when evaluating any condition. Third, because overweight and obese patients were categorized into a single group, we were not able to assess differences in clinical manifestations between overweight FMS patients and obese patients separately. A larger sample size would be needed to perform such analysis.

In summary, we found that overweight/obesity was associated with several clinical manifestations of FMS including self-reported memory impairment and urinary frequency in this group of Puerto Ricans with FMS. Because overweight status and obesity are extremely prevalent comorbidities in FMS patients, these findings, together with other similar reports, may provide useful clinical data when assessing, treating, and counseling FMS patients. Moreover, these observations aim to the need of conducting prospective studies to evaluate this relationship in more detail and to further elucidate the role of BMI in FMS and further determine the impact of weight reduction interventions on the occurrence or severity of FMS-related manifestations.

## Figures and Tables

**Table 1:** Sociodemographic characteristics and health-related behaviors in nonoverweight/obese and overweight/obese patients with fibromyalgia syndrome.

Feature	Normal and underweight BMI ≤ 24.9 (*n* = 35)	Overweight and obese BMI ≥ 25 (*n* = 109)	*p* value
Age, mean years (SD)	50.2 (10.1)	50.2 (9.9)	0.986
Gender, % female	100	93.6	0.136
Education (≤12 years), %	14.3	26.6	0.135
Exercise, %	25.7	24.8	0.911
Cigarette smoking, %	5.7	6.4	0.621
Alcohol drinking, %	0.0	0.9	0.755
Disease duration, mean years (SD)	4.5 (4.3)	5.0 (4.9)	0.565

BMI: body mass index; SD: standard deviation.

**Table 2:** Clinical manifestations in nonoverweight/obese and overweight/obese patients with fibromyalgia syndrome.

Feature	Normal and underweight BMI ≤ 24.9 (*n* = 35)	Overweight and obese BMI ≥ 25 (*n* = 109)	*p* value
*Current symptoms*, %			
Tiredness	94.3	97.3	0.352
Anorexia	22.9	22.0	0.917
Weight loss	14.3	7.3	0.179
Insomnia	71.4	66.1	0.555
Headache	74.3	73.4	0.917
Anxiety	65.7	82.6	0.035
Depression	68.6	78.0	0.259
Memory impairment	62.9	82.6	0.015
Arthralgias	100.0	94.5	0.182
Joint swelling	31.4	44.0	0.187
Myalgias	94.3	91.7	0.622
Morning stiffness	85.7	86.2	0.938
Shortness of breath	42.9	62.4	0.042
Constipation	54.3	56.0	0.862
Diarrhea	28.6	27.5	0.904
Urinary frequency	31.4	61.5	0.002
Paresthesias	71.4	85.3	0.063
Raynaud's phenomenon	22.9	19.3	0.645
Sicca symptoms	71.4	68.8	0.770
Dysmenorrhea	33.3	53.7	0.163
*Physical examination*			
Tender points, median (IQR)	16 (12–18)	18 (14–18)	0.043

BMI: body mass index; SD: standard deviation. IQR: interquartile range.

**Table 3:** Comorbidities in nonoverweight/obese and overweight/obese patients with fibromyalgia syndrome.

Comorbidity	Nonoverweight/obese BMI ≤ 24.9	Overweight/obese BMI ≥ 25	*p* value
	(*n* = 35)	(*n* = 109)	
	%	%	
Osteoarthritis	48.6	62.4	0.148
Cervical spine disease	34.3	27.5	0.444
Lumbar spine disease	31.4	42.2	0.257
Osteoporosis	11.4	8.26	0.391
Peripheral neuropathy	17.4	20.2	0.693
Hyperthyroidism	0.0	0.92	0.757
Hypothyroidism	11.4	14.7	0.434
Hypertension	28.6	37.6	0.330
Hyperlipidemia	40.0	44.0	0.675
Type 2 diabetes mellitus	0.0	12.8	0.016
Coronary artery disease	0.0	1.8	0.572
Stroke	0.0	1.8	0.572
Bronchial asthma	11.4	16.5	0.334
Chronic obstructive pulmonary disease	0.0	0.9	0.757
Anemia	2.9	8.3	0.250
Malignancy	0.0	3.7	0.321

**Table 4:** Current and past pharmacological and nonpharmacological therapies in nonoverweight/obese and overweight/obese fibromyalgia patients.

	Current therapy			Past therapy		
	Nonoverweight/obese	Overweight/obese	*p* value	Nonoverweight/obese	Overweight/obese	*p* value
Therapy	BMI ≤ 24.9	BMI ≥ 25		BMI ≤ 24.9	BMI ≥ 25	
	(*n* = 35)	(*n* = 109)		(*n* = 35)	(*n* = 109)	
	%	%		%	%	
Tricyclic antidepressants	5.7	18.4	0.055	37.1	48.6	0.236
Selective serotonin reuptake inhibitors	28.6	22.0	0.427	57.1	65.1	0.394
Serotonin-norepinephrine reuptake inhibitor	34.3	44.0	0.309	57.1	66.1	0.340
Anticonvulsants	31.4	53.2	0.025	65.7	72.5	0.444
Benzodiazepines	14.3	25.7	0.163	22.9	42.2	0.040
Muscle relaxants	28.6	28.4	0.988	68.6	70.6	0.816
Nonselective nonsteroidal anti-inflammatory drugs	28.6	31.2	0.770	85.7	80.7	0.505
Cyclooxygenase-2 inhibitor	14.3	11.9	0.713	54.3	51.4	0.764
Opioids	0.0	3.7	0.324	5.7	15.6	0.108
Psychotherapy	22.9	32.1	0.298	45.7	48.6	0.764
Physical therapy	2.9	9.2	0.201	71.4	69.7	0.514
Acupuncture	2.9	0.9	0.428	20.0	13.8	0.261
Chiropractic therapy	2.9	0.9	0.428	20.0	11.0	0.141

**Table 5:** Fibromyalgia manifestations independently associated with overweight/obesity by logistic regression analysis.

	Multiadjusted^*∗*^ OR (95% CI)	*p* value
Memory impairment	3.35 (1.27–8.86)	0.015
Anxiety	2.49 (0.91–6.80)	0.076
Shortness of breath	1.93 (0.81–4.63)	0.139
Increase of urinary frequency	2.96 (1.23–7.13)	0.015
Tender points	1.03 (0.948–1.124)	0.465

^*∗*^Adjusted for gender, age, type 2 diabetes mellitus, disease duration, current use of anticonvulsant, and past use of benzodiazepines; OR: odds ratio; CI: confidence interval.
